# Late chronic total occlusion of infrarenal aortic stent graft and iliac branch device presenting as chronic limb-threatening ischemia

**DOI:** 10.1016/j.jvscit.2026.102348

**Published:** 2026-06-10

**Authors:** Kazumasa Hanada, Ayako Nishiyama

**Affiliations:** Department of Vascular Surgery, Saiseikai Kawaguchi General Hospital, Kawaguchi, Saitama, Japan

An 83-year-old man was admitted to our department with a diagnosis of right-sided chronic limb-threatening ischemia (CLTI), staged as Rutherford classification 5 with a nonhealing ulcer and rest pain of the first toe, and gastric cancer. His medical history included endovascular aortic repair with a left-sided iliac branch device [GORE EXCLUDER AAA C3 and iliac branch endoprostheses; W. L. Gore & Associates)] and right internal iliac artery coil embolization performed 76 months earlier for aneurysms measuring 36 mm in the abdominal aorta, 35 mm in the right common iliac artery, 35 mm in the right internal iliac artery, and 45 mm in the left common iliac artery. The patient provided consent for publication of his clinical details and images.

Computed tomography performed 3 months before admission for bilateral calf claudication revealed total stent graft thrombosis, extending from the juxtarenal aorta to the terminal external iliac arteries. Blood flow to the bilateral common femoral arteries was maintained via the collateral pathways from the internal thoracic arteries to the inferior epigastric arteries (∗) and from the lumbar arteries to the deep circumflex iliac arteries (†), indicating chronic occlusion (A). Bilateral ankle pressure was unmeasurable. Although direct oral anticoagulation was initiated because the patient declined revascularization, anemia resulting from gastric cancer, diagnosed after a bleeding episode, exacerbated the ischemia and led to CLTI.

Laparoscopic distal gastrectomy was performed, taking care to preserve those collaterals. Thirty days later, a right axillobifemoral bypass was performed, which improved CLTI (B). The patient was discharged on postoperative day 7.

Preserving at least one internal iliac artery during the repair of iliac artery aneurysms is recommended.^1^ However, total occlusion of the stent graft after a combination of unilateral iliac artery aneurysm embolization and contralateral iliac branch device placement may cause pelvic and lower limb ischemia. Although thrombotic occlusion of stent grafts typically presents as acute limb ischemia, its presentation as CLTI is uncommon.^2^^,^^3^ In the present case, the combination of proximal stent graft protrusion and progressive aortic tortuosity over time led the minor curvature to occlude the aortic lumen, resulting in total stent graft occlusion. This chronic process allowed development of the collateral circulation, a vital pathway present in >95% of patients with aortoiliac occlusive disease^4^; however, anemia secondary to gastric cancer caused CLTI.

**Figure:**
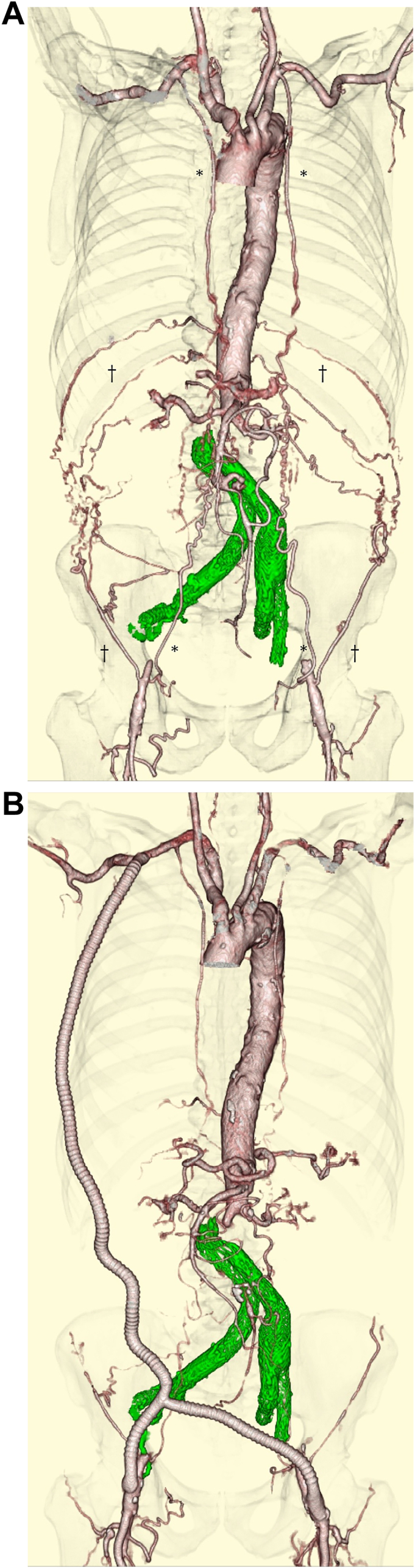

## Funding

None.

## Disclosures

None.

## Figures and Tables

**Cover image:**
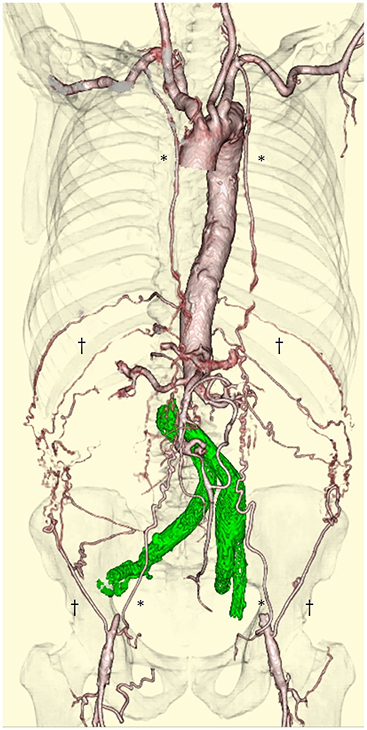


## References

[1] Chaikof E.L., Dalman R.L., Eskandari M.K. (2018). The Society for Vascular Surgery practice guidelines on the care of patients with an abdominal aortic aneurysm. J Vasc Surg.

[2] Daye D., Walker T.G. (2018). Complications of endovascular aneurysm repair of the thoracic and abdominal aorta: evaluation and management. Cardiovasc Diagn Ther.

[3] Rathore A., Gloviczki P., Oderich G.S., Bower T.C. (2019). Collapsed bifurcated modular infrarenal endograft. J Vasc Surg.

[4] Yurdakul M., Tola M., Ozdemir E., Bayazit M., Cumhur T. (2006). Internal thoracic artery-inferior epigastric artery as a collateral pathway in aortoiliac occlusive disease. J Vasc Surg.

